# Thermal, Morphological, Electrical Properties and Touch-Sensor Application of Conductive Carbon Black-Filled Polyamide Composites

**DOI:** 10.3390/nano11113103

**Published:** 2021-11-17

**Authors:** Valentina Brunella, Beatrice Gaia Rossatto, Domenica Scarano, Federico Cesano

**Affiliations:** NIS (Nanostructured Interfaces and Surfaces) Interdepartmental Centre and Department of Chemistry, University of Torino, Via P. Giuria 7, 10125 Torino, Italy; valentina.brunella@unito.it (V.B.); beatricegaia.rossatto@unito.it (B.G.R.); domenica.scarano@unito.it (D.S.)

**Keywords:** carbon black, polymer composites, polyamide 66, thermal and electrical properties, differential scanning calorimetry, scanning electron microscopy, electrical conductivity, atomic force microscopy, conductive-AFM, capacitive touch sensor

## Abstract

Polyamide 66 (PA66) is a well-known engineering thermoplastic polymer, primarily employed in polymer composites with fillers and additives of different nature and dimensionality (1D, 2D and 3D) used as alternatives to metals in various technological applications. In this work, carbon black (CB), a conductive nanofiller, was used to reinforce the PA66 polymer in the 9–27 wt. % CB loading range. The reason for choosing CB was intrinsically associated with its nature: a nanostructured carbon filler, whose agglomeration characteristics affect the electrical properties of the polymer composites. Crystallinity, phase composition, thermal behaviour, morphology, microstructure, and electrical conductivity, which are all properties engendered by nanofiller dispersion in the polymer, were investigated using thermal analyses (thermogravimetry and differential scanning calorimetry), microscopies (scanning electron and atomic force microscopies), and electrical conductivity measurements. Interestingly, direct current (DC) electrical measurements and conductive-AFM mapping through the samples enable visualization of the percolation paths and the ability of CB nanoparticles to form aggregates that work as conductive electrical pathways beyond the electrical percolation threshold. This finding provides the opportunities to investigate the degree of filler dispersion occurring during the transformation processes, while the results of the electrical properties also contribute to enabling the use of such conductive composites in sensor and device applications. In this regard, the results presented in this paper provide evidence that conductive carbon-filled polymer composites can work as touch sensors when they are connected with conventional low-power electronics and controlled by inexpensive and commercially available microcontrollers.

## 1. Introduction

In recent years there has been an increasing interest in the application of polymer composites. These materials have also been designated to replace metallic components, due to some key advantages, including low cost, lightness, corrosion resistance, high durability and strength [1,2]. Among polymers, polyamides (PAs) represent a large class of engineering thermoplastic macromolecules and when they are in the form of fibres, films, or other shapes they can be used in many applications, including cloths, packaging materials and structural components [3,4,5,6]. 

The addition of suitable fillers to polymers can also improve certain characteristics, giving rise to new and advanced materials. These results can be obtained by combining the typical properties of macromolecules with filler characteristics, including electrical and thermal conductivities, mechanical strength, wettability, optical properties [7,8,9,10]. In this regard, it is widely accepted that properties of the polymer composites are mainly controlled by the filler loading, the microstructure and the interaction at the interface region [11,12,13]. 

With the discovery of new carbon nanostructures, fullerenes (0D), carbon nanotubes (1D) and graphene (2D) have received a huge interest from the scientific community. Such low-dimensional carbon materials are widely used in polymer composites not only to reinforce and improve mechanical properties, but also to make thermally and electrically conductive the typically insulating polymers [10,14,15]. Although the electrical properties of carbon fillers are not comparable to those of metals, except for some doped graphene, CNT films and yarns [16], there are many advantages to fabricate electrically conductive composite materials based on carbon nanofillers for the current and new technologies. Among the most relevant applications, there are: (i) the housing of electrical and electronic parts and other high-voltage applications [1,17], (ii) low-power electronics [16,18,19,20], (iii) piezoresistive and touch sensors [18,21,22,23], (iv) electromagnetic interference (EMI) shielding [24], and (v) antistatic composite materials [25]. 

Notwithstanding all these achievements, CB has been recently rediscovered. On the one side, it is not toxic, easy to be dispersed in solvents, and it is an extremely cheap material (1€/kg) that exhibits excellent electrical conductivity. On the other side, its branched (nano)aggregates can agglomerate to form hierarchical structures that may find application in the fabrication of electrodes, sensors and biosensors [26].

As far as the electrical properties of carbon-filled PA composites are concerned, there is abundant literature. In general terms, the most widely used carbon fillers to be melt compounded with polyamides are CBs, CFs and graphites, which can be processed by extrusion or injection molding techniques [27,28,29]. For example, fabrics made of polyamide-66 (PA66) or polyamide-12 (PA12) containing CB with electromagnetic shield properties for protecting the human body against radiation [30] or thermoplastic nanocomposites with high mechanical resistance and electrical conductivity [14] were fabricated by melt mixing. In other papers, Espera Jr. et al. [31] and Hong et al. [32] used PA12 and CB via a selective laser sintering process for the 3D printing or to obtain composite materials with conductive segregated networks. These examples are representative of more innovative applications of these materials. 

Herein, polyamide 66 was used as polymer matrix and melt-compounded with CB. Crystallinity, thermal properties, morphology, and DC electrical conductivity were investigated for elucidating the role played by the different filler quantities. Furthermore, conductive-AFM mapping of CB/polymer composites made it possible to locally visualize aggregated filler particles in the polymer matrix working as effective conductive paths. A shrewd interpretation of the results and of the adopted method may contribute to the use of such conductive composites as sensors and devices. In this regard, carbon-filled polymer composites were tested as touch sensors connected with conventional low-power electronics and controlled by inexpensive and commercially available microcontrollers.

## 2. Materials and Methods

### 2.1. Materials

Polyamide 66 (PA66) Technyl^®^ A 205 F Natural from Solvay was used as polymeric material. Its characteristics are suitable for injection molding. Carbon black (CB, ENSACO^®^ 250 G supplied by Imerys), is characterized by surface area of 65 m^2^/g, oil absorption number (OAN) of 190 mL/100 g and primary particles with sizes of 40 ÷ 60 nm arranged in three-dimensionally branched particle chains, which have sizes of up to several hundred nanometers [33]. The size of the primary particles, the surface area, OAN value and the type of aggregation classify this particle type as high structure CB. CB powder and polymer were melt-extruded at 270–290 °C (Leistritz 27E co-rotating twin-screw extruder with a diameter of 27 mm and length/diameter of 40:1, respectively) and injected in a mold at 80 °C. The final compounds were obtained in the form of small plates with dimensions of 50 × 50 × 2 mm. 

### 2.2. Methods

Thermogravimetric (TGA) analyses were performed with a Q500 TA Instruments, by increasing the temperature from RT to 700 °C under nitrogen (N_2_) gas flow, then to 800 °C in air (heating rate 10 °C/min in both steps). This method was adopted to determine the polymer and carbon contents after compounding. 

Differential scanning calorimetry (DSC) measurements were performed with a Q200 TA Instruments under N_2_ gas flow, setting up a heat-cool-heat cycle method from 30 to 300 °C (heating rate of 10 °C/min), in order to erase first the previous thermal history of polymeric phase and then to characterize the material properties [34]. A quantity of 11 mg or 3–8 mg were used for TGA or DSC experiments, respectively.

Morphology of melt-compounded samples was investigated by focused ion beam-scanning electron microscopy (FIB-SEM, TESCAN S9000G instrument). Before SEM analyses specimens were cryo-fractured in liquid nitrogen [13]. 

Atomic force microscopy (AFM) was used also to correlate the surface topography with the electrical properties in conductive-AFM (C-AFM) mode. The AFM instrument (Nanosurf EasyScan2 AFM instrument), equipped with a 10 μm scan-head, was placed in an acoustically shielded enclosure (Faraday cage) and on a high-performance anti-vibration platform. Cross-sections of samples with a thickness of 1–1.5 mm were obtained by cutting the compounds perpendicularly to their main direction. The specimen surfaces were ground and polished, while the backside was contacted with a conductive silver paste. The sample was connected to a 10 kΩ variable resistor to limit the current, and a potential of ±0.1 ÷ 1 V was applied between the tip and probe in static mode.

Electrical properties were obtained by using a conventional two-point probe technique connected with a digital multimeter (Keithley 2420 source meter). Each molded plate was cut to obtain a smaller specimen about 20 × 14 × 2 mm. Each face of the specimen was polished to eliminate the outermost surface. Electrical resistivity (ρ) values were obtained by using the equation: ρ = *R* × (*A*/*l*), where *R* is the measured resistance, *A* is the cross-section of the sample and *l* is the distance between the electrodes. Hence, obtained values were referred to as bulk conductivity. Then Ag paste was used on both sides of samples to make electrodes, which were connected with Cu wires working as connecting leads. Measurements were obtained by placing the specimen in a homemade sample holder consisting of a fixed side and a spring-loaded electrode.

CB-filled PA compounds (50 × 50 × 2 mm in size) were tested in a touching/not-touching cycling operations when connected with Arduino Uno (open-source board based on the ATmega328P microcontroller). Ag paste was used to connect the composite specimens with the low-power electronics. A thin-film pressure sensor (RP-S40-ST, DF Robot) was placed on the plates during touching tests, after weight calibration in the 1–1000 g range. The flexible thin-film sensor, optimized to sense static and dynamic pressure at a high response speed, works on the principle that different applied loads give rise to a change in electrical resistance (the output resistance decreases as pressure increases). During touching tests, the voltage drop between the sending pin and the receiving pin was also monitored by a bench multimeter. 

## 3. Results and Discussion

### 3.1. Thermogravimetric (TGA) and Differential Scanning Calorimetry (DSC) Analyses

**Figure 1:**
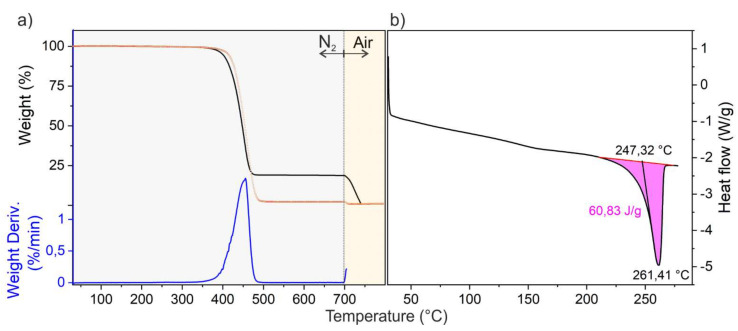
Thermograms of the PA CB-17 composite material: TGA and DTGA plots obtained under N_2_ (up to 700 °C) and air flow (from 700 °C up to 800 °C) (black and blue curves, respectively) (**a**) and DSC analyses (only the second heat cycle is reported) (**b**). In (**a**) TGA plot of pure PA66 is shown for comparison (red dotted line).

TGA and DSC measurements were performed to determine the polymer and filler percentages, melting temperature and enthalpy of fusion of the melt compounded samples. Because of the similarity of the great majority of the thermograms, the curves shown in Figure 1 are to be considered as representative of the whole series of measurements. 

In particular, from Figure 1a,b, TGA and DSC thermograms of the PA-CB 17 sample (PA66 filled with 17 wt. % CB) are shown. In detail, the weight loss percentage, together with its derivative by weight percentage and the heat flow as a function of the temperature, is shown in Figure 1a,b, respectively. It can be observed that at a lower temperature (320–520 °C interval), degradation of the polymeric matrix occurs, while at a higher temperature (700–800 °C) the burning of carbon fillers takes place. 

In order to calculate the percentage of filler in composite materials, the sample coded PA Technyl (pure PA66) was analyzed for reference (Figure 1, grey curve). Under an inert flow, pure PA66 degrades by 97 wt. %. From the weight loss of the TGA curves, the percentage of polymer inside each composite in the same temperature range (320–520 °C) can be evaluated and by difference, the CB percentage.

The results of the investigated samples are summarized in Table 1. 

From these values, it is clear that the melting temperature of the specimens is not remarkably affected by the addition of filler. Enthalpy of fusion and consequently crystallinity of CB-filled samples were found to be lower than that of neat PA66. In this regard, the crystallinity decreases by enhancing the quantity of incorporated CB, according to what was observed for semicrystalline polymers (e.g., PA, PP, PLA), whose crystallization is affected by the presence of additives [36,37,38,39]. 

### 3.2. Scanning Electron Microscopy (SEM) Analysis

**Figure 2:**
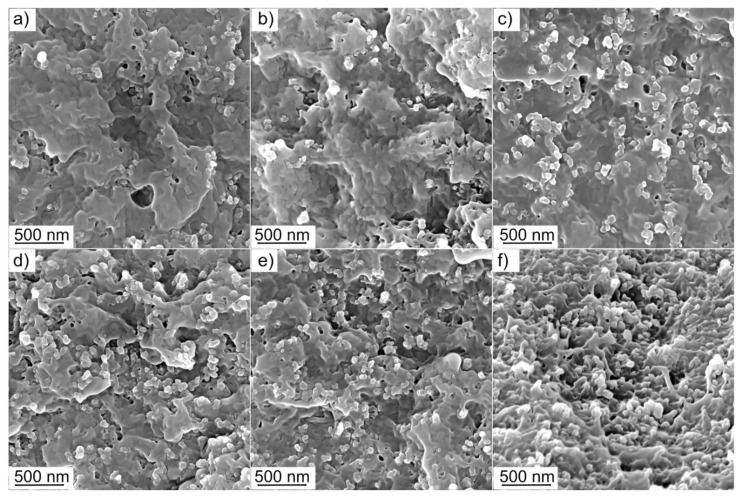
SEM images of CB-reinforced PA66 melt compounded composites with different filler content: 14 % (**a**), 16 (**b**), 17 (**c**); 18 (**d**); 21 (**e**) and 27 wt. % (**f**), respectively.

In Figure 2a–f, cross-sectional SEM images of melt-compounded samples with different CB loadings (14, 16, 17, 18, 21 and 27 wt. %) are shown. 

**Figure 3:**
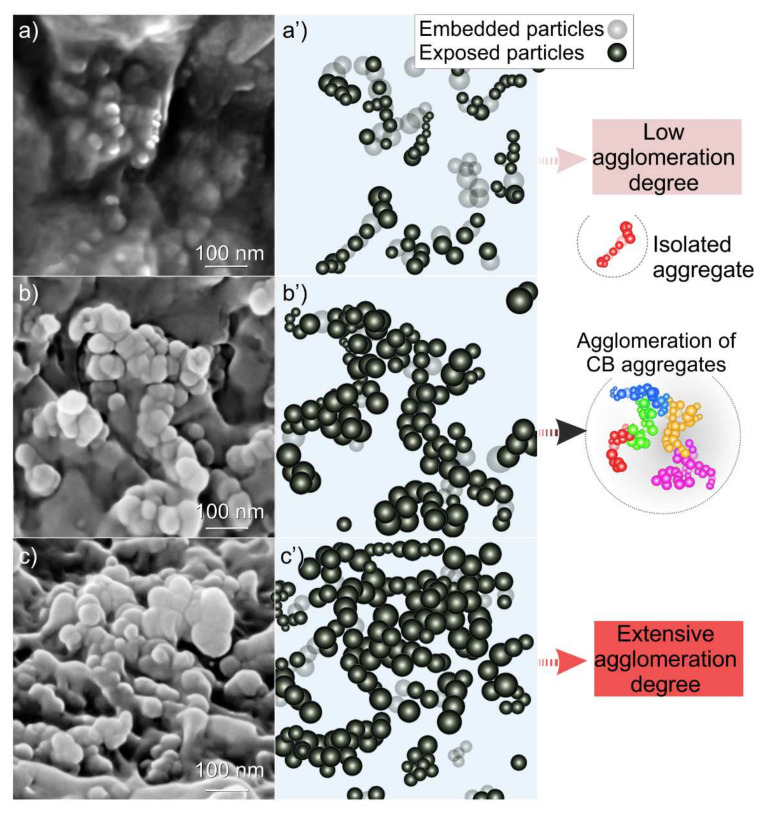
High-resolution FESEM images of CB-reinforced PA66 melt compounded composites with different filler content: 14 % (**a**), 17 (**b**), 21 wt. % (**c**), and the associated schematic representations illustrating the CB particles, their aggregates at the different filler loadings: 14 (**a’**), 17 (**b’**), and 21 wt. % (**c’**); respectively.

By comparing these images, the different filler quantities together with the dispersion degree and microstructure of CB, can be highlighted. The dispersion degree of the carbon filler depends on its nature and content inside the polymer matrix. In particular, high-structure CB aggregates of various sizes and shapes [40] forming dense networks inside the polymer matrix, cannot be evidenced in Figure 2. Higher magnification images were obtained on specimens containing 14, 17 and 21 wt. % of CB (Figure 3a–c). 

In these images at the lower CB amount (CB 14 wt. %, Figure 3a) CB aggregates appear to be more confined and isolated, but form agglomerates at the increased CB content becoming larger and more branched as the CB content increases from 17 to 21 wt. % (Figure 3b,c). These comments are even more remarkable if a model representing the microstructures of the CB for the three different compositions is taken under consideration (Figure 3a’–c’). 

Remarkably, the agglomerated CB aggregates shown in Figure 3 are also very similar in appearance to the high-structure CB before melt-compounding, as obtained from TEM images [41].

### 3.3. DC Electrical Properties at 25 °C

**Figure 4:**
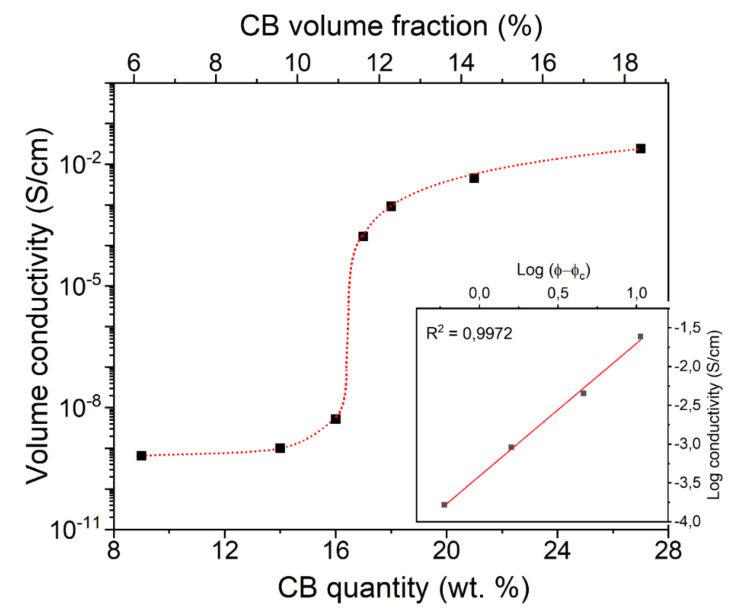
Room temperature electrical conductivity as a function of the CB loading in CB-filled melt-compounded PA composites. The log–log plot of conductivity at 25 °C is shown in the inset.

The DC electrical properties of the CB-filled polymer composites at room temperature are shown in Figure 4. In this figure, the insulator-to-conductor transition is observed near 16.5 wt. % (11.7 vol%) of CB. Above this quantity, known as critical filler concentration, the electrical percolation threshold is exceeded, and the measured DC electrical conductivity of the composite specimens abruptly jumps up to five orders of magnitude, as the formation of continuous conducting paths in the material is achieved.

The DC conductivity *σ* was assessed by the power-law relationship based on the following conventional percolation theory: *σ* = *σ*_0_ (*φ* − *φ_c_*)*^t^*, where: *σ_0_* is a scaling factor, *φ* represents the filler percentage, *φ_c_* is the filler percolation threshold, and *t* is a critical exponent. *φ_c_* and *t* were determined via a least-squares fitting of the log of conductivity plotted versus log (*φ − φ_c_*) and the values were varied up to the best linear fit (inset of Figure 4). The above equation is well fitted by the experimental data for *φ* > *φ_c_*, with φ_c_ = 16.5 wt. % and *t* = 1.71.

The small increment of electrical conductivity from 21 to 27 wt. % of CB can be explained, considering that the electrical percolation is achieved. Furthermore, the filler aggregates are expected to be preferentially localized in the amorphous regions of semicrystalline-polymers [42] (in fact, CB is more uniformly dispersed in the matrix of amorphous polymers [43]). In this regard, by adding CB beyond this critical point, the conductivity will not be remarkably affected by the amorphous phase distribution [44], due to the fact that the resulting percolation paths are defined. 

In conclusion, the electrical properties of the samples can be explained with a classical percolation approach, in which the electrical percolation threshold is controlled by the necessary connections between conductive CB particles for the occurrence of continuous conductive paths [45]. This explanation appears to be well represented by the FESEM images. It is worth mentioning that the degree of crystallinity (Table 1) decreases with the increased quantity of CB according to the fact such filler is expected to be mainly located in the amorphous phase of semicrystalline polymers (i.e., PA).

### 3.4. AFM Imaging of the Conductive Paths in the Composite Materials

**Figure 5:**
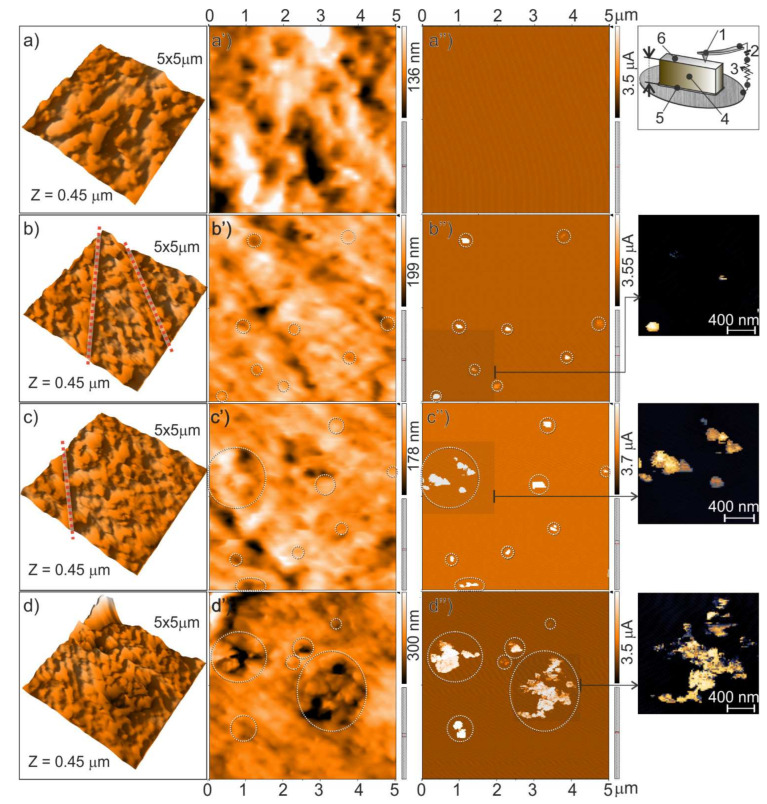
5 × 5 µm AFM images and current maps of CB/PA66 polymer composites with different CB content: 9 (**a**–**a’’**), 17 (**b**–**b’’**), 21 (**c**–**c”**), and 27 wt. % (**d**–**d’’**), respectively; 3D AFM images: (**a**–**d**), related topographic signals: (**a**–**d**), as compared to current maps: (**a’’**–**d”**), respectively, acquired in C-AFM mode. In the insets, schematic representation of the setup for simultaneous topography/current mapping (top inset; 1: conductive cantilever/probe, 2: DC V source, 3: resistor; 4: sample, 5: Ag paste, 6: polished surface. The thickness of the specimen is c.a. 1–1.5 mm) and topography/current mixed signals in selected areas of (**b”**–**d”**), are also illustrated.

It is widely recognized that the SPM techniques enable morphological acquisitions from micro to sub-nanometer scale resolution [46,47]. Contact-mode AFM technique, operating with a DC current from the tip to the sample surface (conductive-AFM), can be adopted for simultaneously acquiring the sample morphology and imaging of conductive pathways in nanofiller-based polymer composites [48,49] and films [50,51]. Electrical transport properties of disordered conductor/insulator domains in such materials are closely associated with the mesostructure (i.e., the aggregation state at the mesoscale) [52], which makes quantitative electrical evaluation difficult in particular at the macroscale level. It is noteworthy that the approach adopted in this work is qualitative and could not demonstrate the percolation through samples at the macroscale. However, the method is simple and effective, allowing to prove the occurrence of percolation paths within small specimens. As discussed in the following, in our experiments the method was used to attain qualitative local electrical properties of the CB-filled composite materials through portions of the composite samples. In this regard, in Figure 5, 5 × 5 µm cross-sectional AFM images of melt compounded CB/PA66 composites with the different CB loadings (9–17–21–27 wt. %) are shown. 

From the reconstructed 3D topographic signals (Figure 5a–d) it is clear that the preparation procedure adopted for the AFM acquisitions is effective in obtaining surfaces with Z-heights suitable for scanning probe techniques. The topographies represent the morphologies of the CB-filled polymer compounds that could be fairly altered by the mechanical polishing (e.g., the two vertical grooves in Figure 5b). In Figure 5a’–d’ the topographic signals of the 9–17–21–27 wt. % CB-filled PA composites are compared, as well as their current maps (Figure 5a”–d”) collected in contact-mode AFM scans under conductive-AFM (C-AFM) acquisitions. The maps were obtained by contacting the tip with a small potential (0.1 ÷ 1 V) relative to the sample backside (Top inset of Figure 5a’’). Conductive regions (except for CB 9 wt. %) are illustrated at the surface with percolation networks passing through the thickness (c.a. 1–1.5 mm) of specimens with composition over the percolation threshold (17–21–27 wt. % of CB). In these AFM conductive maps, together with some bumps (red dotted lines in Figure 5b,c), the majority of conductive zones is associated with small surface tips, as a result of the selective removal of the polymer phase from the surface during the polishing preparation. 

A comparison of the composites with different CB content shows that the conductive areas for the samples with lower CB content (17 wt. %) were more localized, whereas they were more extended (i.e., more extensive CB agglomeration) for the samples with the higher CB content (21 and 27 wt. %), justifying their better electrical characteristics. The comparison of AFM images with topography and current mixed signals (bottom insets in Figure 5) in selected regions of (b”–d”) better highlights at the nanometric scale that the conductive paths are caused by extensive CB agglomeration occurring fairly above the percolation threshold. 

The method described above allows to connect clearly and unambiguously the morphology with electrical properties, also highlighting the impact of the preparation process on the electrical properties. Furthermore, the role played by the agglomeration occurring at the higher CB content, exceeding the electrical percolation threshold, is also emphasized. 

### 3.5. Touch-Sensor Prototype

**Figure 6:**
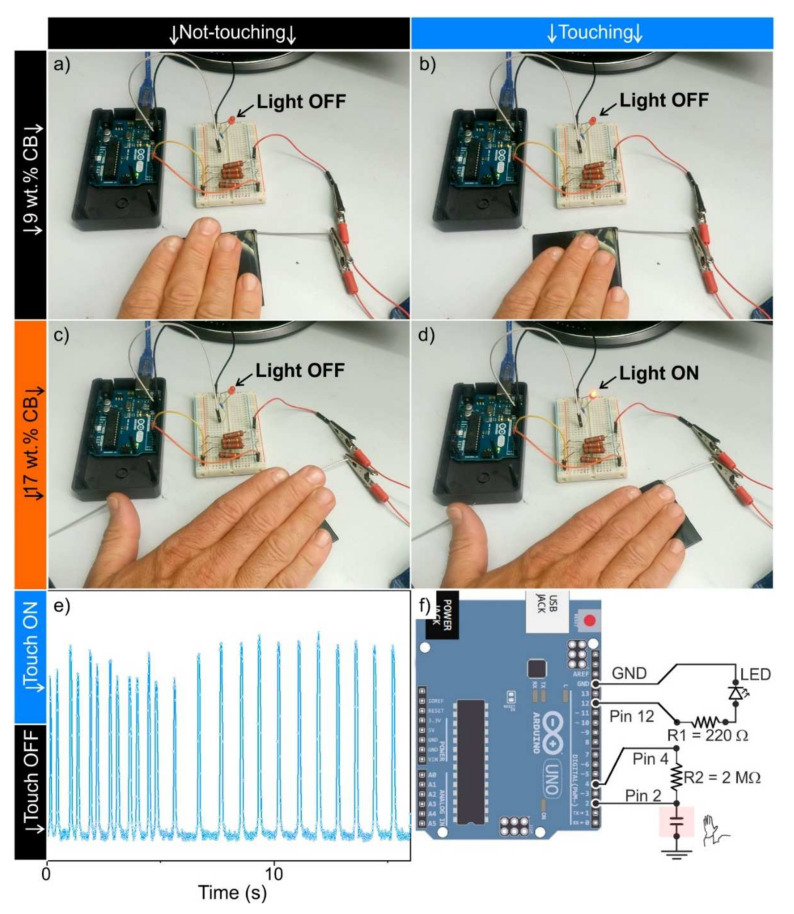
Proof of concept of the melt-compounded CB-filled PA66 specimens (50 × 50 × 2 mm in size) tested under not-touching/touching operations. Compounds containing: 9 (**a**,**b**) and 17 wt. % of CB (**c**,**d**) (i.e., below and over the percolation threshold, respectively); touch sensing output from the device under ON/OFF touching cycles for 17 wt. % of CB (**e**); representation scheme of the Arduino board integrated with the touch sensors (input) and the LED (output) (**f**).

Carbon filler-based polymer composites have recently found application in the field of sensors, including pressure and temperature monitoring systems [18,23,53]. Among the advantages of all-carbon sensors, there are several benefits including lightweight, recyclability, environmental stability, flexibility, low-cost, and small energy consumption. Furthermore, they can be easily connected with traditional electronics [18,54], although the interface with metals (i.e., electrodes) could play a crucial role in operating circuits [55]. In this domain, owing to the different electrical properties, it was decided to test the performance of carbon-based polymer compounds in touch-sensing operations. The I/0 pins of the Arduino Uno board connected with composite plates (50 × 50 × 2 mm in size) of different CB loadings (9, 17, 21 and 27 wt. %) were tested under cycles of not-touching/touching operations. In Figure 6a–d, finger touching for 9 and 17 wt. % CB-filled compounds are illustrated.

The images shown in Figure 6 are to be considered as representative of the whole series: non-conductive (below) and conductive (beyond) the electrical percolation threshold, respectively. Interestingly, the qualitative identification of the finger touch was obtained. The composite specimens with low-conductivity, lower than the percolation threshold, were not responsive to touch (Figure 6a,b, and Supplementary Materials). Conversely, there was touch sensitivity (Figure 6c,d) for high conductive specimens. More interestingly, the touch-sensing output coming from the sensing device under ON/OFF touching cycles of different duration was precisely responding, as illustrated in Figure 6e. From this figure, a fast and positive sensitivity to touch is observed for compounds with filler loadings above the percolation threshold. We can justify it with the conductive network formation, made of one or more electrically conductive paths formed through the specimen, which works as a whole touch-responsive sensor when connected with the I/O pins an Arduino Uno board, as depicted in Figure 6f. However, from this figure the working principle cannot be figured out.

**Figure 7:**
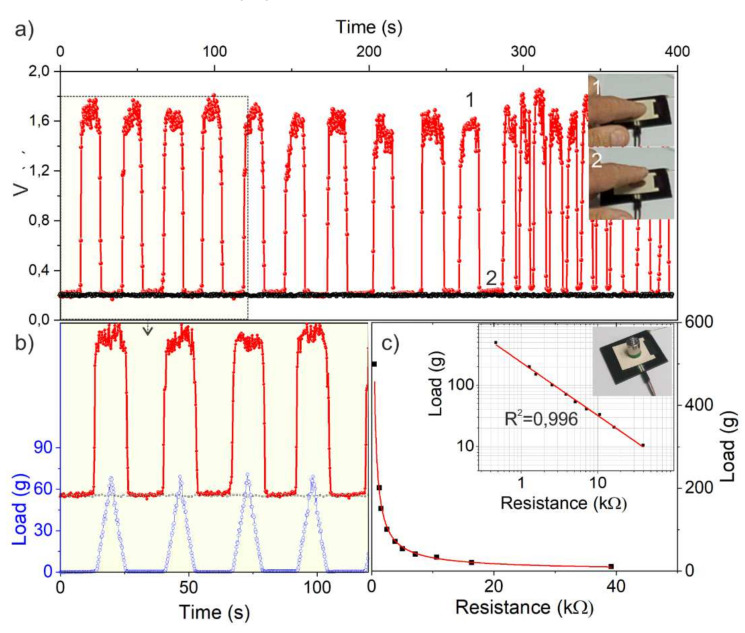
Output voltage between the two I/0 pins of the Arduino board connected with CB-filled PA66 specimens (50 × 50 × 2 mm in size) 9 and 17 wt. % (black and red data points, respectively). In (**a**), 1 and 2 refer to finger touching and not touching events illustrated in the insets, respectively; (**b**) a detail of the output voltage during the finger touching/not touching cycle (red curve: CB 17 wt. %, gray curve: CB 9 wt. %, which was measured together with the load applied by the finger; (**c**) electrical resistance calibration curve vs. the applied loads for the thin film sensor laying on the surface of the composite specimens. In the inset, the log–log plot of the weight calibration curve is shown.

In order to shed light, the voltage drop (V) between the two digital pins was measured in other experiments during the touching cycles for the different CB-loaded PA composite specimens working as sensors (Figure 7a and Supplementary Materials). Remarkably, a potential of c.a. 1.4–1.8 V was abruptly measured during the finger touching (Step 1 in Figure 7a), while it was steady at c.a. 0.2 V without touching (Step 2 in Figure 7a). On the other hand, no significant V output was observed for specimens with electrical properties below or around the electrical percolation threshold during the touching operations (Figure 7a, black data points), thus highlighting the remarkable role played by the different electrical properties. 

In the touching experiments, the applied load by the finger touching was also monitored by a thin film sensor laying on the surface of the polymer composite specimens (Figure 7b), after the sensor calibration of the electrical resistance with applied reference weights (Figure 7c). Interestingly, the significant V drop was measured only during the touch (1.6 ± 0.25 V) and not during the finger approaching (0.2 ± 0.02 V), while no quantitative correlation with the applied load was observed. It is worthy to mention that the sensitivity, intended as the ability to correctly identify touch from no touch events, is very precise for CB-filled compounds with good electrical properties (CB amount ≥ 17 wt. %), while no sensitivity was observed for specimens near or below the electrical percolation threshold. It is also worthy of consideration the fact that the repeatability and reproducibility of the experiments were affected by environmental changes, such as the different ambient temperature and humidity, and other noise sources such as the presence of electrical equipment close to the sensor. Further investigation is needed to account for the different environmental conditions to compensate the baseline employing a more elaborate sensing algorithm.

The explanation for the touch responsivity can be rationally found in the different electrical properties of composite specimens. An electrically conductive composite can behave as a charge accumulator (capacitor) when the device is connected with an oscillating RC circuit such as the digital pins on an Arduino board.

## 4. Conclusions

The present study gives insights into carbon black (CB), one of the most common conductive nanofillers, when it is melt-compounded with polyamide (PA66) to fabricate polymer composites below or above the percolation threshold with 9–14–16–17–18–21–27 wt. % of CB. Morphology, phase composition, crystallinity, thermal behaviour as well as DC electrical conductivity were investigated by using microscopies (FESEM and AFM), thermal analyses (TGA and DSC) and electrical conductivity investigations. Moreover, DC electrical measurements and conductive-AFM mapping through sample portions made it possible to image the CB tendency to form aggregates working as electrically conductive paths above the electrical percolation threshold. The texture properties of CB aggregates and the occurrence of the high filler loadings of larger agglomerates inside the polymer matrix were also investigated by adopting microscopy methods. Shrewd interpretation of the relationship between the quantity of CB and of the obtained electrical properties also contributes to enabling the use of such conductive composites in sensor and device applications. Interestingly, conductive CB-filled polymer composites, with CB loading beyond the electrical percolation threshold, work as touch sensors when they are connected with conventional low-power electronics and controlled by inexpensive and commercially available microcontrollers. Owing to the microstructure of the composite, when the bulk of the compound is electrically conductive, the entire specimen responds without any sensitivity to position, when connected with simple control electronics. These concepts can also be interpreted and extended to other types of conductive carbonaceous fillers to be used in compounding for metal-free low-power sensing technology. 

## Figures and Tables

**Table 1:** Composition, melting point, enthalpy of fusion and degree of crystallinity (%), as obtained from TGA and DSC measurements.

Sample Name	Filler Content (wt. %)/Type ^1^	Melting Point (°C)	Enthalpy of Fusion (ΔH) Measured (J/g)	Degree of Crystallinity (%) ^2^
PA Technyl	-	263.3 °C	67.0	30
PA-CB 9	9/CB	262.1 °C	66.6	29
PA-CB 17	17/CB	261.4 °C	60.8	27
PA-CB 21	21/CB	261.7 °C	57.4	25
PA-CB 27	27/CB	263.0 °C	47.6	21

^1^ CB: carbon black; ^2^ degree of crystallinity (%) = (measured ∆H/100% crystallinity) × 100. If entirely crystalline, samples of PA66 have ΔH of 226 J/g [35].

## Data Availability

The data presented in this study are available on request from the corresponding author.

## Supplementary Materials

The following are available online at https://www.mdpi.com/article/10.3390/nano11113103/s1, Supplementary Video: CB-filled PA compounds connected with Arduino Uno board: a touch sensor prototype.
